# Polyandry as a mediator of sexual selection before and after mating

**DOI:** 10.1098/rstb.2012.0042

**Published:** 2013-03-05

**Authors:** Charlotta Kvarnemo, Leigh W. Simmons

**Affiliations:** 1 Department of Biological and Environmental Sciences, University of Gothenburg, Gothenburg, Sweden; 2 Behavioural and Evolutionary Ecology Group, Section of Environmental and Marine Biology, Department of Biosciences, Åbo Akademi University, Turku, Finland; 3 Centre for Evolutionary Biology, School of Animal Biology, University of Western Australia, Crawley, Western Australia, Australia

**Keywords:** Bateman gradient, direct and indirect benefits, mating competition, sex roles, sperm competition, variation in reproductive success

## Abstract

The Darwin–Bateman paradigm recognizes competition among males for access to multiple mates as the main driver of sexual selection. Increasingly, however, females are also being found to benefit from multiple mating so that polyandry can generate competition among females for access to multiple males, and impose sexual selection on female traits that influence their mating success. Polyandry can reduce a male's ability to monopolize females, and thus weaken male focused sexual selection. Perhaps the most important effect of polyandry on males arises because of sperm competition and cryptic female choice. Polyandry favours increased male ejaculate expenditure that can affect sexual selection on males by reducing their potential reproductive rate. Moreover, sexual selection after mating can ameliorate or exaggerate sexual selection before mating. Currently, estimates of sexual selection intensity rely heavily on measures of male mating success, but polyandry now raises serious questions over the validity of such approaches. Future work must take into account both pre- and post-copulatory episodes of selection. A change in focus from the products of sexual selection expected in males, to less obvious traits in females, such as sensory perception, is likely to reveal a greater role of sexual selection in female evolution.

## 1. Introduction

**Figure 1.**
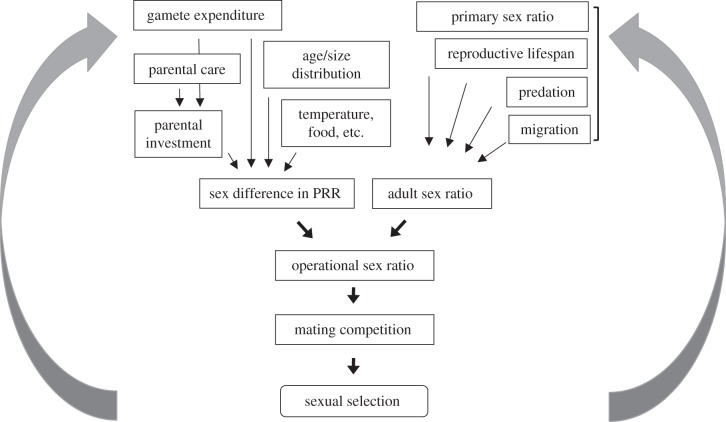
Operational sex ratio (OSR) is a central concept for understanding variation in mating competition and sexual selection within and between species. OSR is the ratio (m : f) or proportion (m/m + f) of males and females that are ready to mate in a population at any one time [4,9]. In many species, both sexes show some level of competition for mating opportunities. However, when the relative number of opposite sex individuals that are ready to mate becomes rare, competition intensifies, and stronger pre-copulatory sexual selection is expected among the sex in excess. The OSR in turn is influenced by the adult sex ratio (ASR) and by a sex difference in potential reproductive rates (PRRs) [5]. All else being equal, PRR is the reproductive rate individuals would achieve if they were given unlimited access to mates. If one sex has a higher PRR than the other, this difference will push the OSR towards the faster sex. A sex difference in PRR can arise due to one sex making a higher parental investment, because of higher expenditure on gametes or parental care. All else is rarely equal, however. An individual's age or size often influences its PRR, and so do ecological factors such as food availability and temperature. Especially in ectotherms such as fish, amphibians, reptiles and insects, low temperature often slows PRR. Whenever these effects on PRR differ between the sexes, they are expected to generate changes in the OSR [9,10]. While effects via PRR are typically delayed, effects via ASR are instantaneous [11]. Naturally, the primary sex ratio affects ASR fundamentally, but many other factors can also affect it, for example if the reproductive lifespan, migration pattern or predation risk differs between males and females. If acquisition of a resource, such as a nest site, is needed before an individual becomes ready (‘qualified’) to mate or breed, the qualified sex ratio should replace ASR in the flow chart [12]. Insofar as individuals benefit from multiple mates, Bateman gradients link mating competition to sexual selection. But this need not always be so, for example if individuals are monogamous, or if they compete for a territory or other resource required to mate, sexual selection can still operate without covariation between number of mates and reproductive success. As shown here, a wide range of factors can influence mating competition and sexual selection via OSR. Naturally, sexual selection can be influenced by many processes that are not included in this illustration. Our intention here is not to be exhaustive, but simply to highlight the diversity of interactions between intrinsic and extrinsic factors that underlie the form and strength of sexual selection.

The products of sexual selection that function as ornaments or armaments are found predominantly, though not exclusively, in males [1]. The fundamental cause of this sexual difference is believed to lie in the greater expenditure on gametes made by males and females [2]. Because males often invest less in any given reproductive event, they should be able to mate more frequently than females so that their reproductive success is more variable, and dependent on their ability to compete successfully for access to multiple females [2]. Numerous quantitative measures of the opportunity for sexual selection have been proposed, including relative parental investment [3], the operational sex ratio (OSR) [4], potential reproductive rates (PRRs) [5] and the variance in mating success [6–8]. Intrinsic and extrinsic factors interact in often complex ways to ultimately determine the extent to which males and females limit each other's reproductive potential (figure 1). We agree with Parker & Birkhead [7], who argue that the slopes of the so-called ‘Bateman gradients’ offer key measures because they can, in theory, indicate directly the intensity with which sexual selection operates. However, we recognize that empirical measurement of Bateman gradients in natural populations of animals is not without its problems, and that caution is necessary in interpreting positive slopes as evidence of sexual selection when other measures of the opportunity for selection, or data on selection differentials for traits under selection, are unavailable [13]. Nonetheless, Bateman gradients have considerable heuristic value for the theme of this volume, because they address explicitly how polyandry will influence sexual selection.

**Figure 2.**
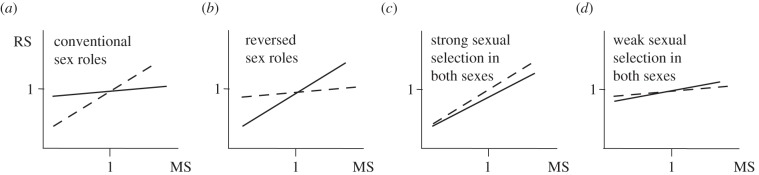
A Bateman gradient (or sexual selection gradient) is the slope of a linear regression between mating success (MS), measured as number of mates, and reproductive success (RS) measured as number of offspring [14,15]. Here standardized Bateman gradients (i.e. with mean set to unity) illustrate four scenarios (*a–d*), with dashed lines for males and solid lines for females. A steeper slope denotes a stronger directional selection for mating success. (*a*) When males have a steeper slope, we expect conventional sex-roles (*sensu* [16], i.e. that males are the sex that predominantly competes for partners) to arise and (*b*) reversed sex-roles (that females are the sex that predominantly competes for partners) when females have the steeper slope. More troublesome when it comes to ‘sex-role’ terminology is the fact that both sexes can show similar directional selection for mating success, with (*c*) steep or (*d*) shallow slopes in both sexes.

Bateman gradients connect mating success, measured as number of mates, to reproductive success. When the slope of the Bateman gradient is steep, one can expect strong selection among individuals for access to as many mates as possible, because the fitness pay-off from multiple mates is high. When males compete more strongly for mating opportunities than do females, males should have a steeper gradient than females (figure 2*a*), as found for some, but not all, of Bateman's fruit flies [14,17]. Conversely, when females compete more strongly than males for mating opportunities, we expect the female gradient to be steeper (figure 2*b*), as found in the sex-role-reversed species of pipefish, *Syngnathus typhle* [18]. Yet, we might also expect both sexes to show steep gradients, such that both benefit from having multiple mates (figure 2*c*), or shallow gradients (figure 2*d*) with neither sex benefitting greatly. Such scenarios illustrate both the general utility of Bateman gradients in predicting mate competition, and why the labelling of competition as a ‘sex role’ obfuscates reality.

In their contribution, Parker & Birkhead [7] show theoretically how considering the costs and benefits of multiple mating can moderate Bateman gradients, and thus the intensity of sexual selection acting on males and females. Polyandry is shown to reduce the difference between male and female gradients, though not which is greater [7]. In our contribution, we draw upon the empirical literature to argue that polyandry can indeed have a major impact on the strength of sexual selection. The Darwin–Bateman paradigm in its original form argued that females gain little from mating with more than one male, predicting that females should have a Bateman gradient that tends to zero. We therefore open this review with a discussion of the selective pressures that can promote the evolution of polyandry and thereby show why female gradients can often be steeply positive (§2). We then discuss how polyandry can influence the strength of competition for access to multiple mates for both sexes (§3), the consequences of polyandry for sexual selection acting after copulation—sperm competition and cryptic female choice (§4), and how these mechanisms of post-copulatory sexual selection can impact net sexual selection operating on males and females (§5).

## 2. Why do females mate with more than one male?

Mating with more than one partner is common among animals. Bateman realized that this represents the principal way by which males increase their reproductive success [2] (figure 2*a*,*c*). It is now clear that female reproductive success can also depend on number of mates, because of direct and indirect benefits, and because of effects of genetic diversity within broods. Indeed, a recent study of the cricket *Gryllus campestris* reported that, in a natural population, the gradient of the relationship between number of mates and lifetime reproductive success was of equal magnitude and positive for both males and females [19] (cf. figure 2*c*).

There are many costs of mating in general, and with multiple partners in particular [20,21]. For example, mate searching is often associated with time, energy and increased predation risk, as is competition for mates, courtship and mating activities [22,23]. The risk of being infected by sexually transmitted diseases and costs related to immune function are likely to increase with number of mating partners [24,25], and mating can be costly for females if male-derived chemicals or physical damage reduces their lifespan, and hence potential lifetime reproductive success. Nonetheless, where the benefits of mating exceed these costs, so that females have positive Bateman gradients, selection is expected to favour the evolution of polyandry. Here, we discuss the benefits females can gain from mating polyandrously.

### (a) Direct benefits

Jacanas provide a well-studied example of how females can increase their reproductive output from having more partners. Both in the wattled, *Jacana jacana*, and bronze-winged jacana, *Metopidius indicus*, a single female can mate with up to four males, laying a clutch of four eggs that each male will incubate [26,27]. Similarly, in the short-snouted pipefish, *Nerophis ophidion*, the female attaches a clutch of eggs to the male's body, which he broods until offspring hatch four to six weeks later [28]. Each male only carries eggs from one female at a time, and because the egg maturation rate of females is considerably higher than the brooding rate of males, each female can, on average, produce two full clutches, while a male broods one. Thus, for both the jacanas and pipefish, the male contributes substantially to offspring care, and although the eggs are large and expensive to produce, female PRR is higher than male PRR. Hence, a female benefits from mating with several males, but only competitively successful females manage to do so. Because females engage in intra-sexual contest competition for mating partners, this leads to sex-role reversal, *sensu* [16], and because large, dominant and highly ornamented females are more successful, it generates both intra- and inter-sexual selection for these traits. Because a larger number of mates translate directly into a higher reproductive success for the females, it also means that species such as these are likely to have Bateman gradients with steeper slopes for females than for males (cf. figure 2*b*). In *N. ophidion*, males father all the offspring they care for [29], despite the polyandry. By contrast, there is a high level of multiple paternity in jacana broods [26]. In bronze-winged jacanas, not all males in a harem get eggs to incubate, but females appear to ‘bribe’ such males to stay by allowing them copulations [27]. If males without a clutch still father some young, this should reduce the variance in reproductive success among males. Still, it is not entirely clear what females gain from having such excess males.

In most animals, females do not double their reproductive success when mating with two males instead of one, like the pipefish and jacana can. Still, they can benefit directly from polyandry in a number of ways. Females that mate with several partners can receive more paternal care and protection for themselves and their offspring [20]. Mating with several males can also increase egg production, owing to nutrients in the ejaculate or nuptial gift [30–32]. Mating with multiple males can secure against infertility, which is important in populations in which infertile males are common [33,34]. Polyandry can also improve female fertility, because mating with multiple males generates a greater supply of sperm [31,35]. This may be particularly important in externally fertilizing species, in which sperm numbers can be severely limiting [36]. Sperm numbers can be limiting for females also in internally fertilizing species, for example if insemination fails [34], or if the most popular males become sperm depleted [37] or allocate sperm strategically [38]. Ironically, while sperm depletion should be less of a problem for monogamous animals, polyandry might be both cause and solution to sperm limitation [39].

### (b) Indirect benefits

Indirect benefits arise when certain genes or combinations of genes raise offspring fitness [20,40], and can be divided into effects that relate to viability or attractiveness [20]. There is ample evidence that mate choice can target both types of genetic quality, and a substantial body of research has investigated the various traits used to signal it [23,40].

Genetic compatibility refers to how the male and female genomes complement each other in ways that affect offspring performance (fertilization, growth, survival, etc.) [41–43]. Again, there is good evidence of mate choice that targets such non-additive genetic effects, or mate choice that targets both ‘good’ and compatible gene effects [42–46]. However, when the genetic quality of mates cannot be determined before mating, females may increase their chances of finding a compatible sire for their offspring by mating polyandrously [41,47]. Recent work suggests the same can be true for males that mate polygynously [48]. In fact, a recent meta-analysis suggests that post-copulatory mechanisms are more likely to generate ‘good’ or compatible genetic benefits than is pre-copulatory female choice based on male secondary sexual traits [39]. Consistent with this, several empirical and theoretical studies have shown that polyandry can evolve in response to genetic incompatibility [49–52].

When polyandry leads to sperm competition, a male's ability to gain successful fertilizations can be related to the relative number of sperm, their ability to reach and fertilize the eggs, displacement of other males' sperm and preventing or delaying female remating. When this competitive edge is heritable, it should result in competitively superior sons [53,54]. Likewise, if a male's fertilization potential is positively associated with his genetic quality more generally, polyandry could deliver viability benefits for offspring produced by superior sperm competitors [55,56]. Experimental evolution studies in house mice have demonstrated how polyandry leads to the evolution of increased early embryo viability [57], and comparative analyses suggest that this effect may be general across mammalian species [58]. In §5, we discuss how these forms of post-copulatory sexual selection can moderate sexual selection operating before mating.

### (c) Genetic diversity and relatedness within broods

Genetic diversity among offspring is lower in broods produced by two parents than in broods produced by three or more contributors, whether it is due to polygyny, polyandry or both. In habitats that are unpredictable from one generation to the next [59,60], increased genetic diversity among offspring may increase the chances that at least some survive [59], in a similar manner to that argued for the evolution of sex [61]. Such effects of polyandry have, for example, been found in an experimental study of the pygmy grasshopper, *Tetrix subulata* [62], and manipulated polyandry has been shown to increase colony resistance to parasites in the bumblebee, *Bombus terrestris* [63]. Restricted circumstances are required for polyandry to evolve as a result of these effects however, such as small population size and low costs of mating with more than one male [59].

Genetic and phenotypic diversity of a brood can also affect competition among siblings. If full-sibs compete more intensely for limited resources than half-sibs, for example owing to more overlapping demands, survival rates should be higher among broods consisting of more diverse offspring [59], as recently shown in *S. typhle* [64] and in the ascidian *Ciona intestinalis* [61]. This effect is likely to be most important if the offspring of a brood are in close proximity, competing for resources before [64] or after birth [65].

Given that polyandry can offer females so many direct and indirect benefits, it is clear that our understanding of sexual selection generally, and our refinement of the Darwin–Bateman paradigm, particularly, needs to consider selection on, and arising from, multiple mating by females.

## 3. Polyandry and mating competition

### (a) Competition among females for mating opportunities

Polyandry can generate competition among females for mating opportunities. As we have seen, in species where females obtain reproductive benefits from males, the female Bateman gradient will be positive, and we should expect to see competition among females for access to multiple males. The intensity of female competition for mates will depend largely on the benefits of polyandry and the costs of mating for males in terms of their time out [5] (or mating latency [66]) from the mating pool. A long time out decreases the PRR. In the bushcricket, *Kawanaphila nartee*, males transfer a large spermatophore at mating, consisting of an ampulla that contains the ejaculate and around it a protein secretion of the accessory glands. The latter serves both as paternity guard, protecting the ampulla from being eaten by the female before the ejaculate is transferred, and as paternal investment, because the proteins consumed by the female contribute directly to offspring production [67,68]. Early in their reproductive season, there is a shortage of pollen-rich flowers, which limits egg production. At this time, females harvest the nutritious spermatophores that males offer [69,70]. Because males also use pollen to produce spermatophores, this means that male PRR is low early in the season, with males taking several days following mating before they can collect enough pollen to be ready to mate again. Elevated female mating frequency coupled with long time out for males generates a female-biased OSR, which causes females to compete for the limited supply of males [71,72].

Female competition in *K. nartee* involves episodes of scramble competition with females racing to locate sexually signalling males, and contest competition with females struggling with already mounted females, attempting to usurp males from those who have arrived first. Gwynne & Bailey [73] found a selective advantage for females with larger auditory spiracles during scramble competition, as this gives females greater auditory sensitivity, allowing them to locate a sexually signalling male rapidly. Moreover, male *K. nartee* appears to promote female competition by producing very short bursts of song that are more difficult for females to locate, compared with the continuous song they produce under high nutrient availability, when males instead compete to attract a limited supply of sexually receptive females [73]. Interestingly, auditory spiracle size exhibits considerable sexual dimorphism in this species, expected of a trait subject to intense sexual selection. In this case, the female has the larger auditory spiracle and the greater auditory sensitivity [74,75].

Female competition for access to males has been widely documented where males or the resources they provide to females are in limited supply [76,77]. However, because of variation in male mate quality, female competition might also be expected when males are not the limiting sex [77]. When females exhibit strong mating preferences, more attractive males, or those of higher genetic quality, may be in greater demand than less attractive males. Under these conditions, females will compete for access to high quality males. For example, there are now a number of studies of birds and mammals in which males form communal display grounds, or leks, which have reported intense female competition over access to the most attractive males [78–80]. In part, this competition might be anticipated because attractive males in high demand from choosy females can become sperm depleted [81,82], selecting for females able to monopolize the attention of attractive males or to mate with these males before they become sperm depleted [77].

It is now clear that polyandry can influence female reproductive success, and that females can be subject to sexual selection. Indeed, there has been a general increase in research and debate on the role of sexual selection in the evolution of female reproductive biology [77,78,83]. Much of the current empirical work is focused on explaining the evolution of relatively rare examples of female ornaments or armaments [84–86], mainly because these kinds of traits are generally recognized to be the products of sexual selection acting on males. Importantly, however, the products of sexual selection via female competition may often be more subtle, as illustrated by *K. nartee*, with sexual selection favouring sensory adaptations in females that allow them to locate males quickly [73]. Similarly, in the common goby, *Pomatoschistus microps*, females respond to increased female competition by laying larger clutches of eggs [87], whereas in *S. typhle*, the presence of competitively superior, large females, causes small females to invest more in growth and less in current reproduction [88], thereby improving future competitive ability.

### (b) Competition among males for mating opportunities

Female mating patterns have long been recognized as influencing the strength of male mating competition [4,40]. In species where females are continually receptive and highly polyandrous, males are often unable to monopolize females, resulting in comparatively low mating skews. In Soay sheep, for example, an increased availability of sexually receptive ewes results in a reduction in the strength of selection on male body and horn size, because when receptive females are in abundance, males are unable to use these traits to control access to multiple females [89]. The monopolizability of females appears to be a major factor influencing mammalian mating systems [90]. Likewise, in dung beetles, horns that are used in the monopolization of mates are less likely to evolve in species that occur in crowded communities, where males are unable to efficiently monopolize the continually receptive females [91].

By contrast, where females mate only once, male competition can be extreme, generating both protandry (where males emerge or reach sexual maturity before females) and extreme mating skew among males. For example, in heliconid butterflies, monandry has resulted in pupal mating [92], whereby adult males copulate with females before they emerge from their pupal case. Likewise, in solitary bees, males emerge often several days before females and competition for access to females as they emerge is intense, with the largest males achieving a disproportionate share of matings [93]. Protandry can also arise under female polyandry, if there is a benefit to the male of mating first, such as higher fecundity in young females [94] or first male paternity advantage, as in the bushcricket *Requena verticalis* [95]. In fact, male *R. verticalis*, which provide a costly nuptial gift, exhibit mate choice, selecting against older females that are less likely to be virgins [95].

## 4. Post-copulatory sexual selection acting on males

Sperm competition [96,97] and cryptic female choice [98] are probably the most widely appreciated consequences of polyandry for sexual selection acting on males. Polyandry can promote the spatial and temporal overlap of ejaculates from multiple males at the time of fertilization, so that sexual selection will favour adaptations in males that ensure their own sperm are successful in fertilizing available ova, and prevent rival males from gaining access to fertilization opportunities. Such adaptations might act via direct competitive interactions between males over paternity, or as signals, delivered during copulatory courtship [98] and used by females in selecting among sperm from prospective fathers. Naturally, post-copulatory sexual selection could act on females as well, but this is a subject that has rarely been addressed [99].

In depth reviews of the evolutionary consequences of post-mating sexual selection for male reproductive behaviour, morphology and physiology are available elsewhere [97,100,101]. Therefore, we provide an overview of these here and explore how increased male investment into adaptations that result from female polyandry can reduce the potential strength of sexual selection acting on males. In §5, we then focus on the potential interactions between pre- and post-mating sexual selection, how post-mating sexual selection might impact the net strength of sexual selection acting on males, and how male expenditure on traits subject to post-mating sexual selection might affect the operation of pre-mating sexual selection via its impact on male PRRs.

### (a) Selection on male reproductive expenditure

A range of male reproductive traits that promote paternity have evolved in response to polyandrous mating by females. Morphological adaptations that enhance paternity are perhaps best documented in the insects, with sexual selection favouring adaptations in male genital morphology that displace or remove sperm of rival males from the site of fertilization, giving the last male to mate a fertilization advantage [102]. Polyandrous females frequently play an active role in the removal or displacement of sperm, responding cryptically to stimuli received from male genitalia during copulation [103,104]. Thus, across insect taxa, species where females are polyandrous tend to have more complex male genital morphology than do monandrous species [105]. Within polyandrous taxa, male and female genital morphology frequently exhibit strong signals of coevolutionary change [106–108].

The degree to which males can displace rival sperm can depend on the time they spend copulating with a given female [97,109]. In the yellow dung fly, *Scatophaga stercoraria*, males do not use their genitalia to remove rival ejaculates. Rather, rival sperm are flushed from the female's sperm store by their own ejaculates, such that the proportion of offspring sired by a copulating male rises with diminishing returns [110,111]. A male's fitness can depend critically on the time he spends copulating, the costs of prolonged copulation in terms of his ability to find and copulate with additional females, and in the case of yellow dung flies the time he spends guarding the female while she deposits her eggs.

A variety of forms of mate guarding have evolved in response to polyandry. Behavioural mate guarding is common across a variety of taxa, including insects [97], birds [101] and mammals [112]. However, mate guarding can also be achieved in ways that do not require the guarding male to be physically present. For example, male insects [97], reptiles [113,114] and mammals [115] deliver secretions to the female reproductive tract that harden to form mating plugs that serve as barriers to copulation by rival males. Among mammals, the protein secretions that form these plugs are under strong sexual selection that increases with the degree of polyandry [116–119]. In *Drosophila*, males transfer seminal fluid proteins within their ejaculates that suppress female sexual receptivity to remating and promote the immediate laying of eggs that will be fertilized by the copulating male [120]. These seminal fluid proteins are likewise characterized by a rapid evolutionary divergence [121] that is dependent on the degree of polyandry [122]. Tettigoniids also transfer compounds within the ejaculate that inhibit female sexual receptivity [69], and comparative analyses have found negative evolutionary covariation between ejaculate volume and the degree of polyandry [123]. Finally, in some insects, males transfer antiaphrodisiac pheromones to females, rendering them unattractive to other males. These pheromones show a faster rate of evolutionary divergence in clades where females are polyandrous [124].

Given that expenditure on mate guarding can have the effect of reducing a male's ability to mate with additional females [125,126], selection is expected to favour phenotypic plasticity in these components of pre- and post-copulatory expenditure to maximize the fitness returns of individual males. Indeed, we see considerable phenotypic plasticity within species, with males investing in mate guarding more heavily when the risk of sperm competition is high. For example, in mammals [127], birds [128], reptiles [129] and insects [130–133], males guard for longer, or with greater intensity, when the risk of female remating with rival males is elevated, whereas in *Drosophila*, males copulate for longer [134] and transfer greater quantities of receptivity inhibiting seminal fluid proteins when in the same situation [135,136]. In many taxa however, males are unable to control female mating frequencies, so that sperm competition conforms to a raffle in which a male's fertilization success depends critically on the number of sperm he has at the site of fertilization relative to his competitors [38].

Implicit in Bateman's [2] argument was the assumption that sperm were cheap, and that males were limited in their reproduction only by the number of females they could acquire. However, we now know that ejaculate production can represent a significant cost of reproduction for males [137,138]. Studies of insects [139], amphibians [140], reptiles [141], birds [142] and mammals [37] have all shown how males can become depleted of sperm and seminal fluid reserves with successive copulations, limiting the numbers of females with which they can mate. Moreover, there is growing evidence to suggest that males face a trade-off between the allocation of resources to ejaculate production and to pre-copulatory competition over access to females (table 1).

**Table 1.** A review of evidence suggesting that males suffer a trade-off between expenditure on gaining matings versus expenditure on gaining fertilizations.

species	evidence	references
acanthocephalan worms	testes mass decreases with increasing sexual size dimorphism (a proxy for the intensity of male contest competition)	[143]
insects		
*Cyrtodiopsis dalmanni*	juvenile hormone application increases eye span at the expense of testes mass	[144]
*Onthophagus nigriventris*	ablation of developing horns in pre-pupae increases testes mass in adults	[145]
*Gnatocerus cornutus*	selection for increased mandible size generates correlated reduction in testes mass	[146]
*Teleogryllus oceanicus*	negative genetic correlation between courtship song structure and ejaculate quality	[147]
*Nauphoeta cinerea*	competitive interactions between males decrease spermatophore size and sperm numbers	[148]
*Panorpa cognate*	negative genetic correlation between attractiveness and ejaculate investment per mating	[149]
*Photinus greeni*	negative phenotypic correlation between attractiveness and competitive fertilization success	[150]
*Hemideina crassidens*	males with large weapons have relatively smaller testes and ejaculate volumes than males with small weapons	[151]
amphibians		
*Crinia georgiana*	negative among population covariation between testes mass and forearm development	[152]
fishes		
*Oncorhynchus kisutch*	breeding coloration negatively correlated with sperm motility	[153]
*Salvelinus alpinus*	negative phenotypic correlation between red spawning coloration, dominance status and sperm density	[154,155]
*Poecilia reticulata*	negative genetic correlation between ornamentation and ejaculate quality	[156]
*Pomatoschistus minutus*	males lacking breeding coloration have testes 4.3 times larger, in absolute terms, than males with breeding coloration	[157]
*Thalassoma bifasciatum*	males with high mating success divert resources towards mate guarding at the expense of gamete production	[81]
birds		
*Chlamydotis undulata*	males with exaggerated courtship displays show rapid deterioration in spermatogenic function compared with males with less elaborate displays	[158]
*Gallus gallus domesticus*	decline in ejaculate quality is associated with success in dominance interactions	[159]
*Malurus melanocephalus*	negative phenotypic correlation between plumage ornamentation and ejaculated sperm numbers	[160]
mammals		
pinnipeds	among harem breeders, testis mass decreases with degree of sexual size dimorphism (a proxy for the intensity of male contest competition)	[161]
*Homo sapiens*	negative phenotypic correlation between voice attractiveness and ejaculated sperm numbers	[162]

**Figure 3.**
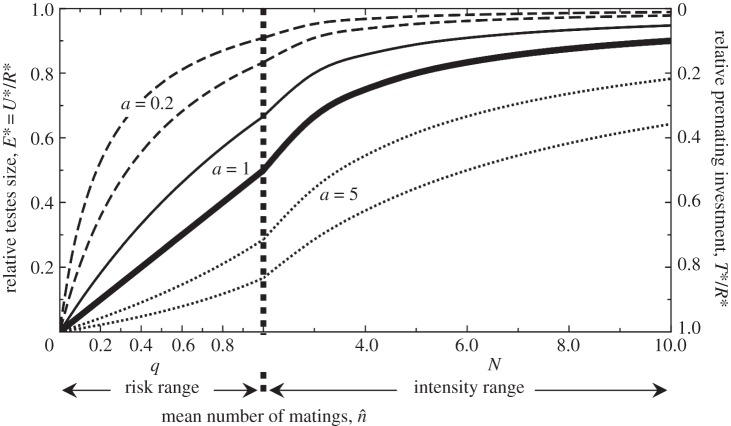
A general model of ejaculate expenditure encompassing the range of male–male competition from contest to scramble. Earlier models used a specific form for pre-mating competition in which a male's number of matings rose linearly with his pre-mating expenditure [38]. However, this formulation includes a parameter *a* that determines the competitive weights of males in relation to their investment in pre-mating competition [152]. When *a* = 1, a male's number of matings rises linearly with his pre-mating expenditure, but when *a* < 1, number of matings increases with diminishing returns, as might occur under mate search. When *a* > 1, male mating success increases more with each increment in pre-mating investment, a situation that might occur under male–male contest competition. The three pairs of curves for values of pre-mating competitive weight 0.2, 1.0 and 5.0 show the predicted change in evolutionarily stable strategy (ESS) ejaculate expenditure (left axis: relative testes size) and ESS pre-mating expenditure (right axis: weapon or signal size) with increasing risk (*q*, probability that a female will mate twice so that there is sperm competition) and intensity (*n*, the number of males mated and thus competing for fertilizations) of sperm competition. The model predicts that for any given risk or intensity of sperm competition, increased competitive weight associated with pre-mating expenditure (*a*) should lead to increased expenditure on male–male pre-mating competition but to reduced ejaculate expenditure. The predictions depend also on the number of males competing for access to each female, *M*. When *M* = 2 (lower curve in each pair), males compete in dyadic contests, but when *M* is very large (upper curve), competition conforms to a scramble. Thus, dyadic contests predict lower ejaculate expenditure and greater expenditure on pre-mating competition. Finally, *M* and *a* are assumed to vary independently, which may not be realistic. For example, as population density increases, the efficiency of male–male contests in securing females may decrease and the cumulative costs increase [163] leading to scramble competition, such that *a* is greatest when *M* = 2 and reduces towards 1.0 or less as *M* increases (from Parker *et al*. [152]).

Mating costs of ejaculate production have recently been incorporated explicitly into Parker's game theoretic models of ejaculate expenditure [152]. Here, males are assumed to have a fixed energy budget for expenditure on reproduction, which can be spent either on acquiring mates (through mate searching, fighting for direct access to females or the resources they require, or courtship displays and ornamentation used to attract females and persuading them to copulate) or on the various ejaculate components that determine male fertilization success (sperm, seminal fluid proteins). Male fitness is assumed to be the product of the number of matings obtained and the fitness gain per mating. Across species, the models predict that males should increase their expenditure on the ejaculate, and decrease their expenditure on gaining additional matings as the risk (probability that a female will mate with more than one male) and intensity (the number of males with which a female mates) of sperm competition increases (figure 3). There is now considerable evidence to support this basic prediction. Thus, across species, increases in the degree of polyandry are associated with increases in testes mass [100,164]. Laboratory evolution studies of single species have also shown increases in male ejaculate expenditure in response to experimentally elevated rates of polyandry, in both insects [165,166] and mammals [167,168], and similar patterns of micro-evolutionary divergence are implicated from cross-population studies of mammals [169], frogs [170] and birds [171] that show covariation in testes size and the degree of polyandry.

As with mate guarding, theoretical models predict that males should adjust their expenditure on gaining fertilizations, depending on the current risks or intensity of sperm competition [38]. Specifically, males should increase their ejaculate expenditure when faced with a risk of sperm competition from rival males, but reduce their expenditure as the number of males competing for a given set of ova increases (sperm competition intensity), because the fitness pay-off per unit of ejaculate expenditure is expected to decline as the number of males competing increases [38]. Studies of mammals [172,173], fish [174,175], birds [176] and insects [177] have all reported increased numbers of sperm ejaculated when males perceive cues to the presence of rival males (sperm competition risk). Indeed, two recent meta-analyses of the literature have reported moderate sized and general effects of the presence of rivals on ejaculate expenditure [178,179]. General support for the prediction that males should decrease their ejaculate expenditure with increasing intensity is perhaps less strong; the general effect size found by Kelly & Jennions [178] was not statistically significant. However, lack of concordance among studies may lie in experimental design. Manipulating a male's perception of the presence of rival males (risk) is simpler than manipulating his perception of sperm competition intensity, because an increased number of males present at the time of copulation may convey cues of heighted risk rather than the number of males actually competing for fertilizations [180]. Studies that have manipulated cues to a female's past mating frequency have provided good support for the prediction that males decrease expenditure with sperm competition intensity [181].

An assumption underlying intensity models is that males adjust their ejaculate expenditure in response to variation in fitness pay-offs for their investment. From a male's perspective, a highly polyandrous female is assumed to be of reduced reproductive value compared with a less polyandrous female. Male responses to sperm competition intensity can, in this sense, be seen as cryptic male mate choice [48]. Male adjustments in ejaculate expenditure have been reported for a number of measures of female quality, including female age, size, fecundity and secondary sexual trait expression [182,183], with larger ejaculates being delivered to females of greater quality. Again, the general effect of female quality on ejaculate expenditure appears both statistically significant and moderate in size, although it varies across taxonomic groups and reproductive mode [178]. These studies show how individual males can exhibit fine-grained phenotypic plasticity in the allocation of their reproductive resources to different females, and that polyandry is a significant driver of male expenditure.

### (b) Impact on male potential reproductive rate and the strength of sexual selection

Adaptations to sperm competition might be expected to affect the strength of sexual selection acting on males insofar as they will influence male fitness via the number of mates acquired, the Bateman gradient. Males that must copulate for extended periods to displace rival sperm or guard their mates from potential rivals must take ‘time out’ from mate searching in order to ensure they fertilize their current female's batch of ova. Likewise, allocation of resources to mating plugs or seminal fluids can be costly in terms of time required to replenish the proteins necessary for additional mating attempts. These energetic costs of sperm competition, which arise as an evolutionary consequence of polyandry, will reduce a male's PRR, the male Bateman gradient [7], and thus the potential strength of sexual selection both among and within species [184,185]. Among species, if males increase their expenditure on the ejaculate with increasing risk of female remating, male time out for gamete and seminal fluid replenishment is likely to increase. This would have the effect of ameliorating any male bias in the OSR, and reducing the opportunity for sexual selection acting via the number of mates males obtain. Within species, as the degree of polyandry increases, the intensity of sperm competition should increase, and as discussed in §4*a*, ejaculate expenditure is then expected to decrease [38,186]. A decreased expenditure on the ejaculate by males would have the counter effect of decreasing male time out, increasing the OSR bias towards males, and thus the opportunity for sexual selection to act via male mating success. Such effects are likely to be strong in species where ejaculate costs are very high. For example, in bushcrickets, males can expend up to 30 per cent or more of their body mass on a single ejaculate, which consists of sperm, seminal fluid proteins that inhibit female receptivity, and the edible mass of accessory gland proteins that females consume during the period in which the ejaculate is transferred from the spermatophore to the female's sperm storage organs [123,187,188]. The magnitude of male expenditure on these large ejaculates comes at a considerable cost, increasing male time out from the mating pool required to replenish spent resources [126]. As we have seen for *K. nartee*, male time out can greatly exceed female time out, generating a complete reversal in the direction of sexual competition, so that females compete for access to males [72,189].

An additional, and probably more widespread, effect of sperm competition on the intensity of sexual selection is expected to arise in species were males provide parental care. Trivers [3] originally recognized that when females are polyandrous, so that their broods contain offspring from multiple males, each male has a lower expected relatedness to the brood, reducing the benefits of paternal care. Subsequent theoretical treatments have concluded that lost paternity through sperm competition can select against paternal care [190–193]. Experimental studies of fish, insects, birds and mammals have revealed how males reduce their expenditure on paternal care when the risk of lost paternity through sperm competition is elevated, although such effects are not always found [194]. A male is expected to respond only to a risk of lost paternity if he can expect to gain greater paternity in future breeding attempts, a factor that might help explain the often reported variation in the effects of paternity on paternal care among species [195]. By contrast, although cause and effect are difficult to distinguish, a broad pattern of negative coevolutionary variation has been found between rates of extra-pair paternity and female dependency on paternal care (estimated from the reduction in female reproductive success when the male is removed) across bird species [196,197]. Simmons & Parker [184] argued that increased polyandry and sperm competition could influence the strength of sexual selection acting on males indirectly, because lost paternity would select for reduced male expenditure on paternal care that would in turn reduce a male's time out from seeking additional mates. Indeed, the theoretical analyses of Kokko & Jennions [195] show that sex roles are predicted by both polyandry and the strength of sexual selection acting on males and females, but not by the initial bias in parental investment into gamete production. Greater sexual selection and female multiple mating were shown to generate more female than male care.

**Figure 4.**
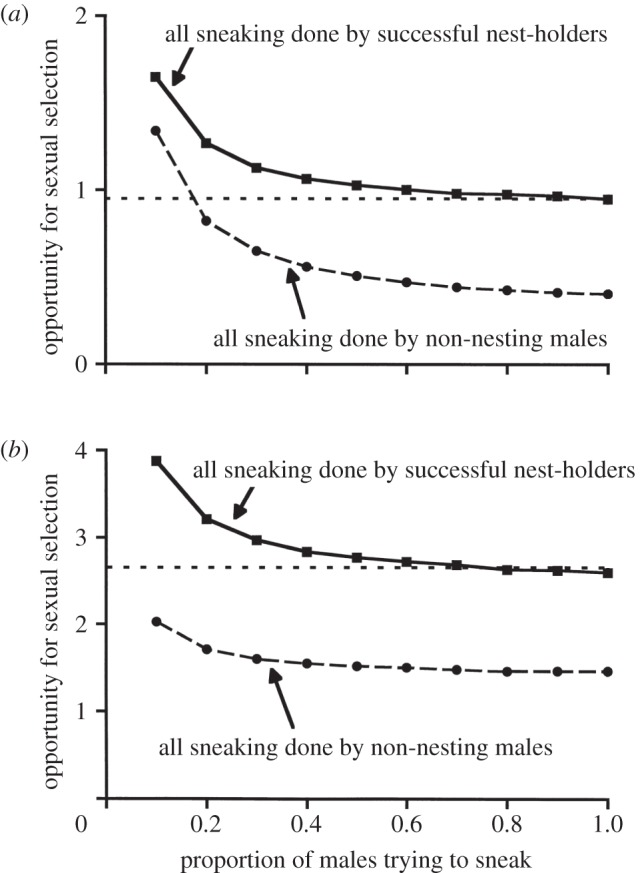
Opportunity for sexual selection related to proportion of males attempting to reproduce parasitically, e.g. through sneaking, based on variance in male mating success when the proportion of unsuccessful (empty) nests is (*a*) 0.425 or (*b*) 0.689. Squares indicate opportunity for sexual selection when all parasitic fertilizations are done by a proportion of nest holders (0.1–1.0). Circles indicate results when all parasitic fertilizations are done by a proportion of non-nesting males (0.1–1.0). Horizontal dashed lines show opportunity for sexual selection without parasitic fertilizations (from Jones *et al*. [204]).

In socially monogamous birds, attractive males who are successful in forming breeding pair bonds are often also successful in gaining extra-pair fertilizations [198–200]. In this manner, sperm competition or cryptic female choice increases the variance in male reproductive success and strengthens the intensity of sexual selection on males [201,202]. However, in polygamous mating systems, lost paternity can dampen the opportunity for sexual selection. In the sand goby, *Pomatoschistus minutus*, males build nests in which they tend batches of eggs from several females. Despite paternal care, sexual selection acts predominantly via male competition for females [203]. Parasitic fertilizations in nest owners' nests are common in this system [204]. Jones *et al.* [204] modelled the opportunity for sexual selection on males, based on the observed variance in male reproductive success estimated from fertilization success within and among nests. They found that the opportunity for sexual selection declined slightly with the proportion of males that took part in sneak matings, but was markedly reduced when parasitic fertilizations were performed by non-nesting males instead of nesting males (figure 4). These contrasting findings for socially monogamous birds and polygynous fishes illustrate how post-copulatory sexual selection can have either synergistic or antagonistic effects on the strength of pre-copulatory sexual selection, a theme we will address in detail in §5.

## 5. Interactions between pre- and post-copulatory sexual selection

**Figure 5.**
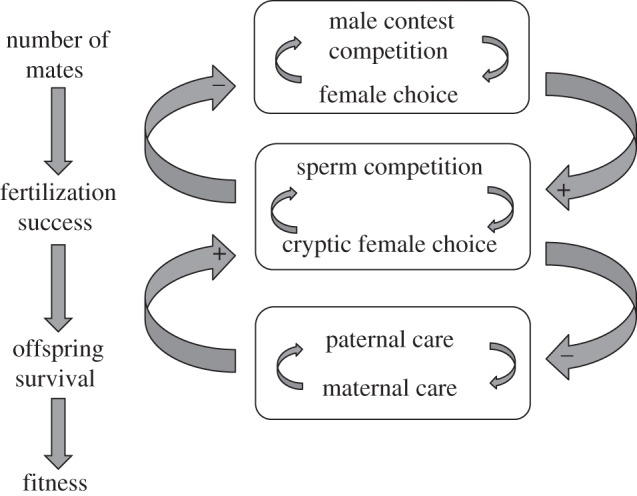
Stages during reproduction at which sexual selection can act. Arrows indicate synergistic (plus symbols) and antagonistic (minus symbols) selection. Opposing selection between male competition and female choice can generate sexual conflict, the resolution of which depends largely on the relative power of each sex to exert their interests over the other, and the value to each sex of winning (see also [206]). The net strength of selection acting on males or females will depend on the interactions between male and female processes within episodes of selection, and on the interactions across episodes of selection.

Quantifying the form and intensity of sexual selection across episodes of male contest competition and female choice has rightly been highlighted as an important endeavour for our understanding of the net force of sexual selection, and thus its evolutionary potential [205]. For example, episodes of male contest competition and female choice can be reinforcing, enhancing the evolutionary potential of that selection. But equally, it can be opposing, so that female choice might ease selection acting via male contest competition. Typically, researchers have estimated the form and strength of selection only at single episodes, rendering our understanding of the evolutionary consequences of selection incomplete [205]. In reality, when females are polyandrous, there are potentially five episodes of sexual selection that need to be quantified; pre-copulatory sexual selection via mate choice and competition for direct access to mates or for resources that qualify an individual to mate [12], and post-copulatory sexual selection via sperm competition and cryptic female choice (figure 5). While there are studies that have examined both male contest competition and female choice operating on the same species [205,207], few studies have extended this into the post-copulatory arena.

Soay sheep, *Ovis aries*, illustrate the ameliorating effects of antagonistic pre- and post-copulatory sexual selection on male fitness. While males compete for access to females, females are highly polyandrous, copulating with as many as 10 different rams per day, which generates intense sperm competition [208]. Data from a free-living population on St Kilda show that body size and horn size independently contribute to male ability to acquire females, and that horn size and testes size independently affect siring success [89]. Importantly, however, intensity of sexual selection acting on male secondary sexual traits depends critically on the availability of ewes, or the OSR. When ewes were limited, pre-copulatory sexual selection was strongest, as expected. However, as the availability of ewes increased, the intensity of post-copulatory sexual selection on testes size increased, and the strength of pre-copulatory sexual selection on body and horn size declined [89]. Moreover, dominant rams that monopolize access to ewes have a high copulation frequency and rapidly become sperm depleted, suffering a reduction in paternity success under sperm competition. Thus, while the copulatory success of large dominant rams increases through the rutting season, their siring success declines, reducing the intensity of pre-copulatory sexual selection acting on male secondary sexual traits [37].

Effects of sperm depletion for males achieving high competitive mating success have also been reported in studies of insect mating systems. In a laboratory study of *Gerris lacustris*, large males were able to obtain a greater proportion of matings [209]. However, small males, when they did obtain a mating, copulated for longer and obtained a higher relative paternity, so that there was no net action of sexual selection on male size [209]. In fireflies, *Photinus greeni*, males with the most attractive bioluminescent displays had the lowest competitive fertilization success [150]. In this experiment, males only mated once. Thus, this probably reflects a trade-off between male expenditure on attractiveness versus competitive fertility rather than on sperm depletion, but the effect is the same, that males favoured in pre-copulatory sexual selection can be disfavoured when females mate polyandrously.

Polyandry may also magnify the effects of pre-copulatory sexual selection. In guppies, *Poecilia reticulata*, females show strong preference for males with large orange patches, male orangeness and female preference show additive genetic variation, and are genetically correlated across the sexes, such that within populations the trait and preference coevolve until checked by natural selection [210,211]. Females also exercise cryptic female choice, accepting greater numbers of sperm from colourful males [212]. Even when female control over insemination is eliminated by artificial insemination of equal numbers of sperm from two males, paternity is biased towards the sperm donor with greater orange coloration [213]. Thus, pre- and post-copulatory sexual selection appear to act synergistically in these fish. Synergistic effects might be expected where high-quality males have enough resources to allocate to both secondary sexual traits and ejaculate production.

Life-history theory is firmly grounded in the notion of trade-offs in resource allocation to fitness related traits [214]. When variance in the allocation of resources required for growth and reproduction exceeds variance in the acquisition of those resources, individuals who invest heavily in attracting females might be expected to have fewer resources available for investment into their ejaculates [215,216]. This is an underlying assumption in sperm competition theory for which there is some good evidence (table 1). However, when variance in acquisition exceeds variance in allocation, we can expect high quality individuals to be in a position to invest heavily in multiple fitness traits [215,216], such as secondary sexual traits and ejaculates [217]. Thus, in some cases, we might expect to see positive correlations between competitive fertilization success and mate acquisition. Indeed, colourful male guppies ejaculate faster swimming and more viable sperm [218], implying that some males can invest heavily in both attractiveness and fertility. Females might thereby ensure good-genes benefits for their offspring not just by pre-copulatory mate choice, but also by polyandry and the competitive fertilization success of good quality males [55]. Where male attractiveness and fertilization success are positively correlated, polyandry is expected to intensify pre-copulatory sexual selection acting via female choice.

The phenotypic expression of traits subject to intense sexual selection via female choice can evolve condition dependence, providing honest signals of the underlying genetic quality of potential mates [40,219,220]. Normally associated with secondary sexual traits, this argument can equally pertain to ejaculate traits that promote paternity [221]. Condition dependence in male fertilization success can thus be an important avenue for cryptic female choice. Evidence for the synergistic action of pre- and post-copulatory female choice comes from dung beetles, *Onthophagus taurus*, in which males court females with a heritable and condition-dependent drumming [222]. High condition males are more attractive, have larger testes and shorter sperm [221]. Unsurprisingly, males with larger testes have a competitive advantage in sperm competition [166]. Moreover, females selectively use the short sperm of high-condition males [223,224]. The consequence of these re-enforcing episodes of pre- and post-copulatory female choice is the production of offspring with enhanced viability [225,226].

Finally, there may be no correlation between pre- and post-copulatory episodes of sexual selection. In a recent study of *D. melanogaster*, fertilization success contributed nearly as strongly as mating success to a male's net lifetime reproductive success. However, males mating last have the fertilization advantage in this species, and when variance in mating order was removed fertilization success explained little of a male's lifetime fitness [227]. The lack of correlation between mating and fertilization success means that measures of mating success alone are inappropriate for estimating overall male success in sexual selection in this population. Such findings are not limited to laboratory studies.

Leks are thought to epitomize sexual selection acting on males, because females mate with only the most attractive male present, resulting in extreme mating skews [228–230], and thus intense sexual selection. However, off lek polyandry can greatly temper the strength of sexual selection. This was found in Houbara bustards, *Chlamydotis undulata*, which at first appear to show a classic lek-based mating system [231]. However, the reproductive skew is nullified via extreme female polyandry [232]. Off lek polyandry is not uncommon in lekking species [80,233,234], questioning the validity of mating success data collected at leks as estimates of the strength of sexual selection.

In general, Parker & Birkhead's [7] simple model predicts that polyandry and sperm competition should reduce the benefits of multiple mates for males, and thus the strength of sexual selection. Indeed, a recent study of feral fowl, *Gallus gallus*, found just such an effect [235]. Polyandry was found to increase the relative importance of post-copulatory episodes of sexual selection on male dominance, relative to pre-copulatory episodes, and simultaneously erode the opportunity for and strength of sexual selection.

The earlier-mentioned studies illustrate how estimates of selection based on mating success alone can provide a limited and sometimes incorrect view of the action of sexual selection. Sperm competition and cryptic female choice can dampen or negate any male advantages obtained via mating success, or can amplify selection, depending on the life history costs of competing for mates and sperm production. There is little reason to expect that there should be any generality here. A growing number of studies are available to show that competitive males can be limited in their ability to produce costly ejaculates and thus unable to capitalize on their mating success (table 1). On the other hand, there are also a growing number of studies that are pointing towards mechanisms capable of generating positive associations between male attractiveness and sperm quality [236–238]. Understanding the net action of sexual selection in a given mating system will require detailed understanding of both pre- and post-copulatory processes.

## 6. Concluding remarks

Our review of the literature illustrates the many ways in which polyandry can moderate sexual selection. In general, when females benefit from polyandrous mating behaviour, for any of the reasons listed in §2, we should expect the slope of the Bateman gradient (translating female mating success to reproductive success) to increase. And as soon as males, or males of high quality, become a limiting resource for females, we can expect females to compete among themselves for such mates. However, polyandry has multiple repercussions for both males and females, before and after mating, and it has several important feedback mechanisms that will impact the action of sexual selection. For example, theoretical models of post-copulatory sexual selection suggest that polyandry will favour the evolution of ejaculate expenditure at a cost of reduced expenditure on ornaments and/or armaments (figure 3). Moreover, polyandry that occurs due to parasitic fertilizations (sneaking, extra-pair fertilizations, cuckoldry, etc.) might be expected to increase sexual selection on males in some cases. However, whether it does or not depends entirely on who gains the parasitic fertilizations—males already successful in gaining multiple females or males that otherwise might not breed [204]. An aspect of post-copulatory sexual selection that we would like to see explored, is selection acting on females via male adjustments in their reproductive investment.

Although a sex difference in the slopes of Bateman gradients, often combined with sexual dimorphism, can be indicative of sexual selection in the more ornamented or armed sex, such sex differences are not required for sexual selection to be acting strongly—*it only makes it easier to discover.* Therefore, the special interest in sex difference in ornamentation [201], Bateman gradients [14] or variance in reproductive success [6], characteristic of much research in this area, carries the risk that we fail to spot less obvious cases of sexual selection. In many cases, both sexes can show steep Bateman gradients, and hence benefit from having multiple mates, or be under strong sexual selection for other reasons.

Historically, sexual selection research has focused strongly on the evolution of male secondary sexual traits, undoubtedly because of the early expectation that female reproductive success should be independent of the number of males mated [2]. However, we now know that polyandry can clearly influence female reproductive success, and that females, like males, are subject to sexual selection. With a general increase in research and debate on the role of sexual selection in the evolution of female reproductive biology [77,78,83], much work has focused on explaining the evolution of female ornaments or armaments [84–86], because such traits are generally recognized to be the products of sexual selection acting on males. Importantly, however, the products of sexual selection via female competition may be less obvious. We believe, therefore, that a shift in focus from the expected products to the process of sexual selection acting on females, will widen our eyes to a far greater role of female competition over access to mates than our current view affords. We also believe that future sexual selection research must consider explicitly the effects of polyandry on mating competition and mate choice for both sexes, and on the net action of sexual selection acting on males and females across both pre- and post-copulatory episodes. Such an approach is essential for a true appreciation of what is arguably the most important agent of evolutionary change.
